# OPTIMISM MITIGATED THE DETRIMENTAL ROLE OF DEPRESSION AND ANXIETY IN SUBJECTIVE WELL-BEING AFTER CARDIAC SURGERY

**DOI:** 10.1093/geroni/igad104.0671

**Published:** 2023-12-21

**Authors:** Amy Ai, Veronika Nash

**Affiliations:** Florida State University, Tallahassee, Florida, United States; Florida State University, Tallahassee, Florida, United States

## Abstract

Cardiac diseases, the primary cause of death among older adults of any races, is comorbid with mental health problems such as depression and anxeity. A recent review article found that cardiovascular health was more consistently associated with optimism and hedonic/subjective well-being than with meaning-laden eudaimonic/psychological well-being. Extensive research has shown the health benefit of dispositional optimism, a personality trait. Few studies, however, have explored the protective effect of optimism the on subjective wellbeing (SWB) one months after cardiac surgery, a life altering procedure in late life. This study estimated the mediating effect of dispositional optimism between preoperative mental health and postoperative SWB. Using a prospective design, we followed 300 patients undergoing cardiac surgery. Mental health was indicated by anxiety and depression, while SWB was indicated by low symptom levels of general distress, based on its definition, one month postoperavely. A confirmatory factor analyses were performed to validate SWB with three subscales. The final solution of a structural equation model (SEM) indicated good fit (Chi-square = 47.55, df = 18, p = 0.000; CFI = 0.95; TLI = 0.92, RMSEA = 0.07, with 90 percent CI from 0.05 to 0.10). Both the CFI (0.96) and TLI (0.93) exceeded the benchmark criterion of .90. The lower CI of RMSEA is under 0.05. Optimism, indicted by two subscales, mediated preoperative mental health and postoperative symptom levels. The finding supports its mitigating role against the damage of depression and anxiety in the SWB of cardiac patients during the critical recovery time – postoperative month.

